# High Glucose-Induced Apoptosis Is Linked to Mitochondrial Connexin 43 Level in RRECs: Implications for Diabetic Retinopathy

**DOI:** 10.3390/cells10113102

**Published:** 2021-11-10

**Authors:** Aravind Sankaramoorthy, Sayon Roy

**Affiliations:** 1 Department of Medicine, Boston University School of Medicine, Boston, MA 02118, USA; asankara@bu.edu; 2 Department of Ophthalmology, Boston University School of Medicine, Boston, MA 02118, USA

**Keywords:** high glucose, apoptosis, mitochondrial connexin 43, endothelial cells, diabetic retinopathy

## Abstract

Diabetic retinopathy (DR) is one of the most common causes of vision loss and blindness among the working-age population. High glucose (HG)-induced decrease in mitochondrial connexin 43 (mtCx43) level is known to promote mitochondrial fragmentation, cytochrome c release, and apoptosis in retinal endothelial cells associated with DR. In this study, we investigated whether counteracting HG-induced decrease in mtCx43 level would preserve mitochondrial integrity and prevent apoptosis. Rat retinal endothelial cells (RRECs) were grown in normal (N; 5 mM glucose) or HG (30 mM glucose) medium for 7 days. In parallel, cells grown in HG were transfected with Cx43 plasmid, or empty vector (EV), as control. Western blot (WB) analysis showed a significant decrease in mtCx43 level concomitant with increased cleaved caspase-3, Bax, cleaved PARP, and mitochondrial fragmentation in cells grown in HG condition compared to those grown in N medium. When cells grown in HG were transfected with Cx43 plasmid, mtCx43 level was significantly increased and resulted in reduced cleaved caspase-3, Bax, cleaved PARP and preservation of mitochondrial morphology with a significant decrease in the number of TUNEL-positive cells compared to those grown in HG alone. Findings from the study indicate a novel role for mtCx43 in regulating apoptosis and that maintenance of mtCx43 level could be useful in preventing HG-induced apoptosis by reducing mitochondrial fragmentation associated with retinal vascular cell loss in DR.

## 1. Introduction

Diabetic retinopathy is a long-term microvascular complication of diabetes. As the diabetic population keeps increasing, the prevalence of DR keeps surging and is estimated at 103 million globally [1]. The Diabetes Control and Complications Trial and the UK Prospective Diabetes Study among others have well-established hyperglycemia to be a major factor in promoting microvascular complications including DR [2]. A prominent early-stage lesion of DR is apoptotic cell loss in the retinal vasculature [3,4,5,6,7]. Since mitochondria and Cx43 are key players in regulating apoptosis, in this study, we investigated the effects of HG on mtCx43 level and how it may impact the development of retinal vascular cell loss in the early stages of DR.

Evidence indicates that mtCx43 plays a critical role in regulating mitochondrial function [8,9,10] and apoptosis mediated through the mitochondrial intrinsic pathway via release of cytochrome c [11,12,13] and initiation of caspase-3 dependent cascade [14]. Several studies have reported the presence of Cx43 in the inner mitochondrial membrane (IMM) [10,15,16,17,18,19], however, their exact function is unclear. Previously we have observed that HG downregulates mtCx43 level concomitant with increased mitochondrial fragmentation and apoptosis in retinal endothelial cells [10]. However, it is unknown whether counteracting the HG-induced decrease in mtCx43 level would be beneficial against HG-induced apoptosis in retinal endothelial cells.

Presence of Cx43 in mitochondria has unveiled certain noncanonical biological processes [20,21,22]. Previously we have shown that downregulation of Cx43 using siRNA in retinal endothelial cells [23] decreases gap junction intercellular communication (GJIC), compromises cell-cell communication and promotes apoptosis [24,25], and that upregulation of Cx43 restores GJIC and reduces apoptosis [10]. Although the role of mtCx43 in regulating mitochondrial dynamics and apoptosis remains unclear, studies suggest that upregulating Cx43 in endothelial cells increases Cx43 level in the mitochondria [15,26,27], which could facilitate preservation of mtCx43 level, inhibition of cytochrome-c release, thereby preventing apoptosis [28]. Additionally, studies have shown that mtCx43 level impacts mitochondrial morphology and apoptosis in cardiomyocytes [8] and that mtCx43 overexpression reduces cytochrome c release and caspase-3 activation [29]. However, it is unknown whether counteracting HG-induced decrease in mtCx43 level would facilitate preservation of mitochondrial morphology, prevent HG-induced mitochondrial fragmentation and thereby prevent apoptosis.

Presence of Cx43 in the mitochondria and the subsequent maintenance of mtCx43 level play a critical role in regulating cell death [30]. Studies demonstrate that the full-length Cx43 is present in the IMM where it is translocated from the cytosol [17] and that inhibition of Cx43 translocation to mitochondria and subsequent reduced mtCx43 level accelerates cell death [16] by reducing cellular respiration [17], promoting mitochondrial energy deprivation and triggering apoptosis [18]. Additionally, evidence suggests that reduced mtCx43 level results in cytochrome c release and apoptosis, indicating that maintenance of mtCx43 level could be useful in preventing apoptosis [14]. However, only limited studies [2] have investigated the effects of HG-induced changes in mtCx43 level in retinal endothelial cells.

Although mtCx43 is involved in regulating mitochondrial morphology [10,27,31], its modulatory role in regulating apoptosis in HG conditions is not fully understood. Given the likely link between mtCx43 level and apoptosis, in this study, we have attempted to counteract HG-induced decrease in mtCx43 level by upregulating mtCx43 expression and investigate whether plasmid-mediated Cx43 overexpression is sufficient to maintain mtCx43 level and thereby prevent HG-induced mitochondrial fragmentation and apoptosis in RRECs.

## 2. Materials and Methods

### 2.1. Cell Culture and Transfection

Rat retinal endothelial cells derived from rat retinas which tested positive for von Willebrand factor (vWF) using immunofluorescence were used in this study as described previously [32]. RRECs were grown in Dulbecco’s Modified Eagle’s Medium supplemented with 10% fetal bovine serum, 100 U/mL penicillin/streptomycin and (Thermo Fisher Scientific, Waltham, MA, USA). To mimic the effect of hyperglycemia RRECs were grown in HG (30 mM) medium for 7 days. In parallel RRECs grown in HG were exposed to pCDNA-Cx43 plasmid (Addgene, Watertown, MA, USA). Transfection of Cx43 plasmid was performed using Lipofectamine 3000 in Opti-MEM (Thermo Fisher Scientific, Waltham, MA, USA) reagent based on the manufacturer’s instructions. DR is a long-term complication of diabetes as such the cell culture model used in this study exposes RRECs to HG (30 mM) for seven days as described [33,34,35].

### 2.2. Western Blot Analysis

The protein expression of cytosolic Cx43, mtCx43, cleaved caspase-3, Bax, and cleaved PARP was determined using 20µg of the total proteins which were separated using a 10–12% SDS-PAGE and transferred onto a PVDF membrane (Millipore, Billerica, MA, USA) using a semidry apparatus (Bio-Rad, Hercules, CA, USA) and blocked with 5% non-fat dry milk for 1h and incubated overnight at 4°C with antibodies against Cx43, Cleaved caspase-3, Bax, and cleaved PARP (catalog numbers 3512S, 9662S, 2774S, 9542S, respectively, at 1:1000 dilution, Cell Signaling Technology, Danvers, MA, USA) antibodies in a solution of Tris-buffered saline containing 0.1% Tween-20 (TTBS) 5% non-fat dry milk. The following day, the membrane was washed with TTBS and exposed to a secondary antibody solution containing AP-conjugated anti-rabbit IgG (Catalog number 7054 at 1:3000 Cell Signaling, Danvers, MA, USA) or anti-mouse IgG (Catalog number 7054 at 1:3000 Cell Signaling, Danvers, MA, USA) for the appropriate primary antibodies, (Catalog number 7056 at 1:3000 Cell Signaling, Danvers, MA, USA) for 1 h at room temperature (RT). The membrane was washed with TTBS, and subjected to Immuno-Star chemiluminescent substrate (Bio-Rad, Hercules, CA, USA) and imaged using chemiluminescence imager (Fuji Film LAS-4000, Tokyo, Japan). The amount of protein loaded in the gel lanes was confirmed through Ponceau-S staining after transfer and exposing the stripped membrane to β-actin antibody (Catalog number sc-47778, at 1:1000, Santa Cruz Biotechnology, Dallas, TX, USA). Protein expression was quantified using densitometric analysis of the chemiluminescent signal at non-saturating exposures and analyzed using ImageJ software [36]. 

### 2.3. Mitochondrial and Cytoplasmic Protein Isolation

Mitochondrial and cytosolic fraction was isolated from RRECs using Mitochondrial isolation from cultured Cells Kit (Thermo, Rockford, IL, USA). Purity of protein samples from cytosolic and mitochondrial cell fractions was determined by probing for voltage-dependent anion channel (VDAC) using antibody against VDAC (Catalog number 4661S, at 1:1000 dilution, Cell Signaling Technology, Danvers, MA, USA). 

### 2.4. Confocal Imaging of Mitochondrial Fragmentation in Live Cells

Confocal imaging was performed using a Zeiss LSM-710 Meta microscope (Carl Zeiss, Oberkochen, Germany) using 63X oil immersion in live RRECs. Cells were incubated at 37 °C in a humidified microscope stage chamber containing 5% CO_2_. Mito Tracker Red (Thermo Fisher Scientific, Waltham, MA, USA) was subjected to 543 nm helium/neon laser excitation and emission was recorded through a bandpass 650 to 710 nm filter (Zeiss, Thornwood, NY, USA). The mitochondrial images were acquired and analyzed using the ImageJ software (version:1.8.0, NIH, Bethesda, MD, USA). The image was first converted to 8-bit and processed using median filter to isolate and equalize fluorescent pixels and particle analysis option to acquire form factor values (FF; (4π*Area/perimeter2)) and aspect ratio (AR), which were calculated from lengths of major and minor axis [37,38]. An aspect ratio value of 1 indicated circular mitochondria, and elongated mitochondria displayed increased AR values. FF value of 1 represents a circular and un-branched mitochondrion. The longer and more-branched mitochondria have higher FF values.

### 2.5. TUNEL Assay

TUNEL analysis was performed using ApopTag Fluorescein Direct in Situ Apoptosis Detection Kit (Millipore, Billerica, MA, USA) according to the manufacturer’s protocol. RRECs were grown on coverslips for 6 days, fixed with 1% paraformaldehyde for 10 min at RT, and permeated for 5min at −20 °C with a precooled mixture of a 2:1 ratio of ethanol/acetic acid. The cells were then washed 2× with PBS for 5 min each, slides were incubated with equilibration buffer and subsequently with TdT enzyme in a moist chamber at 37 °C for 1 h. The cells were again washed with Phosphate buffered saline (PBS) and incubated with anti-digoxigenin peroxidase for 30 min at RT. Finally, cells were washed with PBS and mounted with reagent (SlowFade; Molecular Probes, Eugene, OR, USA). Images of 10 random fields were captured using a digital fluorescent microscope (Eclipse E600; Nikon Corp., Tokyo, Japan) and recorded for analysis. Merged DAPI and FITC stained cells were counted using the Image J software [36].

### 2.6. Statistical Analysis

Statistical analysis was performed using GraphPad Prism software version 9.0 (GraphPad Software, San Diego, CA, USA). All data are expressed as mean ± standard deviation (SD). Values of the control groups were normalized to 100%, and values from all other groups were expressed as percentages of control. Statistical analysis was performed using the normalized values. Comparisons between groups were performed using one-way ANOVA followed by Bonferroni’s post-hoc test. A level of *p* < 0.05 was considered statistically significant.

## 3. Results

### 3.1. HG-Induced Decreased mtCx43

**Figure 1:**
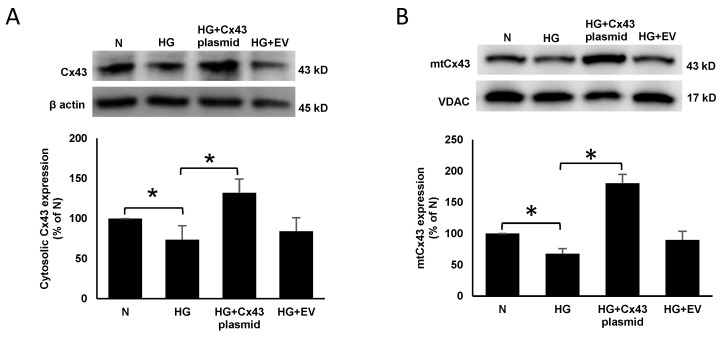
Cx43 plasmid transfection increases mtCx43 level in RRECs. Cytosolic Cx43 expression was significantly decreased in cells grown in HG medium compared to those grown in N medium. (**A**) Cells grown in HG and transfected with Cx43 plasmid significantly increased cytosolic Cx43 expression compared to those grown in HG medium. Bar graph shows cumulative data obtained from six experiments. (**B**) Cells grown in HG and transfected with Cx43 plasmid significantly increased mtCx43 level compared to those grown in HG medium. Bar graph shows cumulative data obtained from six experiments. Data are presented as mean ± SD, * *p* < 0.05; *n* = 6.

WB results indicated a significant decrease in cytosolic (74 ± 17% of N; *p* < 0.05; Figure 1A) and mtCx43 (72 ± 10% of N; *p* < 0.05; Figure 1B) expression in cells grown in HG compared to those grown in N medium. RRECs grown in HG medium and transfected with Cx43 plasmid showed a significant increase in cytosolic (132 ± 17% of HG; *p* < 0.05; Figure 1A) and mtCx43 expression (181 ± 13% of HG; *p* < 0.05; Figure 1B) compared to those grown in HG medium.

### 3.2. Cx43 Overexpression Reduced Bax, Cleaved Caspase-3 and Cleaved PARP 

**Figure 2:**
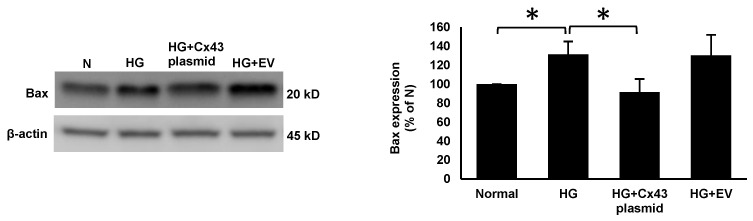
Cx43 upregulation reduces Bax expression. Bax expression was significantly increased in cells grown in HG condition compared to those grown in N medium. In cells grown in HG and transfected with Cx43 plasmid, Bax expression was significantly reduced compared to those grown in HG medium. Bar graph shows cumulative data obtained from six experiments. Data are presented as mean ± SD, * *p* < 0.05; *n* = 6.

**Figure 3:**
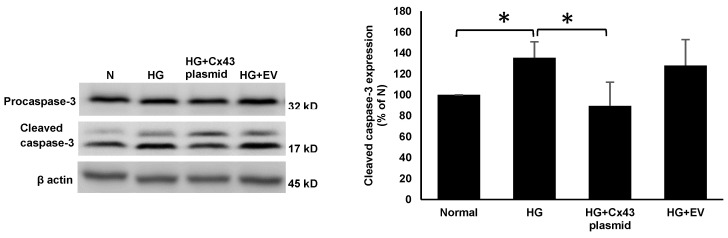
Cx43 overexpression reduces cleaved caspase-3 level. Cleaved caspase-3 level was significantly increased in cells grown in HG compared to those grown in N medium. In cells grown in HG and transfected with Cx43 plasmid, cleaved caspase-3 level was significantly reduced compared to those grown in HG medium. Bar graph shows cumulative data obtained from six experiments. Data are presented as mean ± SD, * *p* < 0.05; *n* = 6.

**Figure 4:**
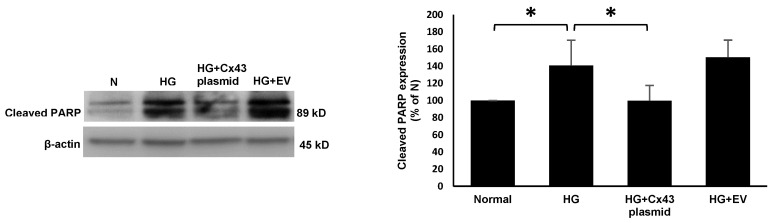
Cx43 overexpression reduces cleaved PARP level. Cleaved PARP level was significantly increased in cells grown in HG compared to those grown in N medium. In cells grown in HG and transfected with Cx43 plasmid, cleaved PARP level was significantly decreased compared to cells grown in HG medium. Bar graph shows cumulative data obtained from six experiments. Data are presented as mean ± SD, * *p* < 0.05; *n*= 6.

RRECs grown in HG medium displayed a significant increase in the expression of Bax (131 ± 13% of N; *p* < 0.05; Figure 2), cleaved caspase-3 (136 ± 15% of N; *p* < 0.05; Figure 3), and cleaved PARP (140 ± 29% of N; *p* < 0.05; Figure 4) compared to those grown in N medium. On the other hand, cells grown in HG and transfected with Cx43 plasmid showed reduced expression of Bax (92 ± 13% of HG; *p* < 0.05; Figure 2), cleaved caspase-3 (106 ± 12% of HG; *p* < 0.05; Figure 3), and cleaved PARP (99 ± 17% of HG; *p* < 0.05; Figure 4) compared to those grown in HG medium.

### 3.3. Cx43 Overexpression Prevented HG-Induced Mitochondrial Fragmentation

**Figure 5:**
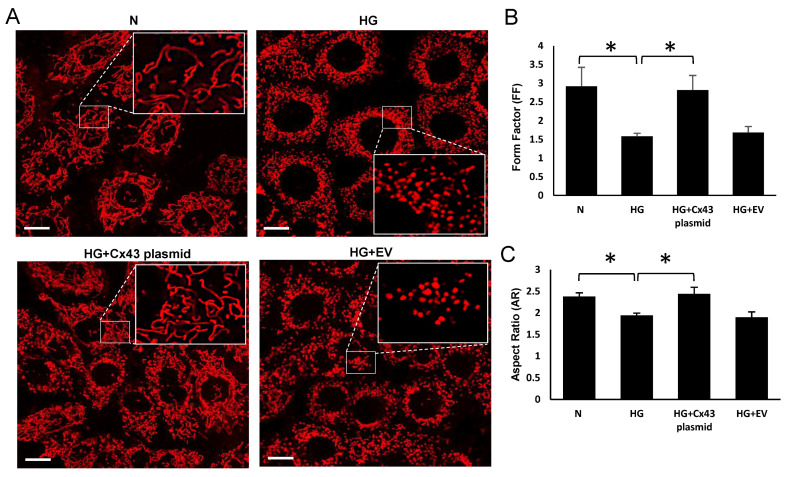
Effect of upregulating mtCx43 level against HG-induced mtCx43 downregulation on mitochondrial fragmentation. Plasmid-mediated Cx43 overexpression improved mtCx43 level and reduced mitochondrial fragmentation in HG condition. (**A**) Representative images of mitochondrial networks in RRECs grown in N medium, HG, HG and transfected with Cx43 plasmid or empty vector, as control. Scale bar = 10μm. The large inset represents an enlarged view of the corresponding field as indicated by dotted lines in each panel. The graph shows average (**B**) FF and (**C**) AR values for mitochondria in RRECs grown in HG medium and transfected with Cx43 plasmid or empty vector. Data are presented as mean ± SD, (* *p* < 0.05; *n* = 5).

Live confocal microscopy of the cells showed increased mitochondrial fragmentation under HG condition; a significant decrease in FF and AR was observed in cells grown in HG compared to those grown in N medium (HG: FF = 1.58 ± 0.12; *p* < 0.05; AR =1.92 ± 0.06; *p* < 0.05; Figure 5B; N: FF= 2.9 ± 0.47; *p* < 0.05; AR = 2.45 ± 1; *p* < 0.05, Figure 5C). The cells grown in HG and transfected with Cx43 plasmid showed significant increase in FF and AR values compared to the those grown in HG (HG+Cx43 plasmid: FF = 2.95 ± 0.47; *p* < 0.05; Figure 5B; AR = 2.51 ± 0.16; *p* < 0.05, Figure 5C). 

### 3.4. HG-Induced Apoptosis Is Prevented by mtCx43 Overexpression

**Figure 6:**
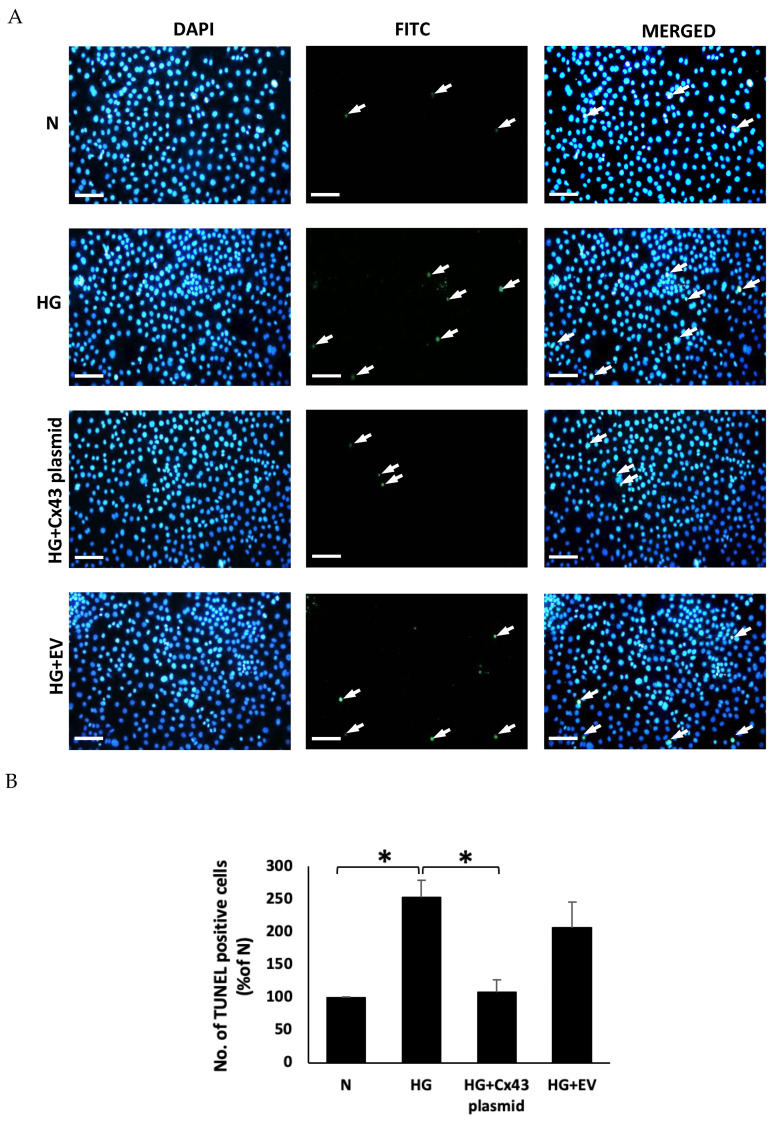
Effect of Cx43 overexpression on apoptosis. (**A**) Representative photomicrographs show a significant increase in the number of TUNEL positive cells (white arrows) in RRECs grown in HG medium compared to those grown in N medium. The cells grown in HG medium and transfected with Cx43 plasmid showed reduced TUNEL positive cells (white arrows) compared to those grown in HG medium. Merged panel shows DAPI stained cells superimposed with TUNEL-positive cells. Scale bar = 100 µm. (**B**) Bar graph shows cumulative data obtained from five experiments. Data are presented as mean ± SD, (* *p* < 0.05; *n* = 5).

TUNEL assay data showed that the number of TUNEL-positive cells was significantly increased in RRECs grown in HG compared to those grown in N medium (254 ± 3%; TUNEL-positive cells compared with TUNEL-positive cells in N; *p* < 0.05; Figure 6A,B). However, RRECs grown in HG and transfected with Cx43 plasmid showed a significant reduction in TUNEL positive cells compared to cells grown in HG (107 ± 2% in Cx43 transfected cells versus 254 ± 3% TUNEL-positive cells in HG; *p* < 0.05; Figure 6A,B), respectively. 

## 4. Discussion

The current study indicates the beneficial effects of increasing mtCx43 expression which prevents apoptosis in RRECs grown in HG condition. Here, we observed that plasmid-mediated overexpression of Cx43 resulted in a significant increase in cytosolic Cx43 level as expected, and importantly, increased mtCx43 level as well. Such increase in mtCx43 level prevented HG-induced mitochondrial fragmentation, reduced Bax overexpression, cleaved caspase-3, and cleaved PARP levels. Findings from this study suggest that mtCx43 upregulation prevents HG-induced mitochondrial fragmentation and apoptosis in RRECs.

The role of mtCx43 in pathophysiological context of diseases including DR is only beginning to be understood. Our previous studies have shown that HG-induced downregulation of mtCx43 in RRECs compromises mitochondrial morphology, increases mitochondrial fragmentation, and apoptosis via release of cytochrome c [10]. Furthermore, Cx43-deficient mitochondria are known to split into smaller fragments, which is indicative of Cx43 as a critical factor impacting mitochondrial fission [39]. In this study, we observed plasmid-mediated Cx43 upregulation in the cytosol increases mtCx43 level in RRECs. However, it is unclear how overexpression of Cx43 in the cytosol results in increased mtCx43 levels. Translocation of Cx43 from cytoplasm to mitochondria is a complex process and likely represents a critical event in regulating mtCx43 level and preserving mitochondrial morphology. Decreased Cx43 translocation resulting in lower mtCx43 level could therefore compromise mitochondrial integrity [40]. Currently, it is unknown whether HG compromises Cx43 translocation to the mitochondria.

Although retinal endothelial cells are well connected to each other via Cx43-mediated gap junctions, non-canonical functions of mtCx43 appear to play a significant role in HG-mediated apoptosis, and possibly in the development of retinal vascular lesions in DR. Evidence suggests that mtCx43 plays a critical role in maintaining mitochondrial dynamics and regulating apoptosis [14] [8]. Studies have shown that inhibition of mtCx43 in brown adipose tissue [40] and rat cardiomyocytes results in reduced mitochondrial integrity, increased cytochrome c release, and increased apoptosis [41,42]. In a chemically-induced model of hypoxia, reduced Cx43 translocation on mitochondria increased mitochondrial ROS production that indicated involvement of mtCx43 in the apoptotic process [43]. Importantly a study has shown that genetic depletion of mtCx43 reverses the protection against ischemia and ROS production [9]. Moreover, reduced Cx43 translocation to mitochondria promotes ROS production [40], which is known to be associated with DR. Future directions to the study would include confirmation of our current findings in an animal model of diabetes and examine whether maintenance of mtCx43 prevents apoptosis.

Overall, findings from the current study demonstrate that maintenance of mtCx43 level plays a critical role in protecting against HG-induced apoptosis in RRECs. This novel approach to regulate and maintain mtCx43 level could thus be beneficial in counteracting the decrease in mtCx43 level under HG conditions, preventing mitochondrial fragmentation, and ultimately inhibiting apoptosis associated with the development of DR.

## Data Availability

Data presented in the article are available by request to corresponding author, Sayon Roy (sayon@bu.edu).
